# Identifying Risk Factors of Postpartum Depressive Symptoms: The Effect of Self-Efficacy and Social Support Among Mothers

**DOI:** 10.3390/healthcare14162608

**Published:** 2026-08-19

**Authors:** Sara A. Alsuhaibani, Atheer F. Alenazy, Leanh S. Alquraishi, Lujain F. Alshaigi, Munirah M. Al Ajmi

**Affiliations:** 1 Department of Health Sciences, College of Health and Rehabilitation Sciences, Princess Nourah bint Abdulrahman University, Riyadh 11564, Saudi Arabia; afalghdeer@gmail.com (A.F.A.); lena.990416@gmail.com (L.S.A.); lujainalshaigi@gmail.com (L.F.A.); imuniiiraalajmi@gmail.com (M.M.A.A.); 2 Ministry of the National Guard-Health Affairs, Riyadh 11426, Saudi Arabia

**Keywords:** postpartum depression, perinatal depressive symptoms, risk factors, self-efficacy, perceived social support, mental health literacy, Saudi Arabia

## Abstract

**Background:** Postpartum depression (PPD) is an important maternal mental health concern. Knowledge, self-efficacy, and social support may be associated with the recognition and severity of depressive symptoms. This study assessed PPD knowledge among pregnant women/mothers and adults from the broader population in Saudi Arabia and examined the associations of self-efficacy and perceived social support with depressive-symptom scores in the maternal group. **Methods:** A cross-sectional online survey was conducted in Saudi Arabia from January to March 2021. Convenience and chain-referral sampling were implemented through WhatsApp. The analytical sample included 432 pregnant women/mothers aged ≥18 years and 275 broader-population participants. Arabic-language questionnaires assessed sociodemographic characteristics and PPD knowledge; the maternal-group questionnaire also assessed depressive symptoms, self-efficacy, and perceived social support. Descriptive statistics, Pearson correlations, linear regression, and one-way ANOVA were used. **Results:** Good PPD knowledge was observed in 73.8% of the maternal group and 77.5% of the broader-population group. Self-efficacy (r = −0.246, *p* < 0.001) and perceived social support (r = −0.197, *p* < 0.001) were negatively correlated with depressive-symptom scores. Both variables were statistically significant in the regression model; self-efficacy had the larger absolute standardized coefficient (β = −0.246) compared with perceived social support (β = −0.197). Depressive-symptom scores also differed across categories of age, family income, education, and previous PPD history. **Conclusions:** The study demonstrated a relatively good level of knowledge about postpartum depression among the surveyed participants. Higher perceived social support and self-efficacy were associated with better maternal mental health outcomes, highlighting their important role in the context of postpartum depression. These findings underscore the relevance of knowledge, social support, and self-efficacy in understanding postpartum mental health among the study population.

## 1. Introduction

Postpartum depression (PPD) is a major public health concern with significant implications for maternal, infant, and family well-being. Globally, it is estimated that PPD affects 10–20% of new mothers, with higher rates ranging from 20 to 40% reported in low- and middle-income countries [1,2]. PPD is defined as a mood disorder that can occur during pregnancy or within the first 12 months following childbirth [2]. Its clinical manifestations include persistent sadness, loss of pleasure in activities, appetite or sleep disturbances, fatigue, feelings of worthlessness or guilt, and in severe cases, suicidal ideation or thoughts of harming the infant [2,3]. This constellation of symptoms can interfere with maternal functioning, affect mother–infant bonding, and negatively influence childhood growth and cognitive development [3].

PPD must be distinguished from related but clinically different postpartum conditions. The “baby blues,” a common and transient emotional state experienced by up to 70% of new mothers, usually emerges within the first 7–10 days postpartum and resolves spontaneously without intervention [4]. Symptoms include irritability, tearfulness, and mood lability, which typically improve with reassurance and social support from family and healthcare professionals [5]. Failure of symptoms to remit may indicate the onset of PPD. Postpartum psychosis, in contrast, is a rare but severe psychiatric emergency affecting approximately 0.2% of women [5]. It is characterized by delusions, hallucinations, severe mood disturbances, and impaired reality testing and often necessitates immediate hospitalization to ensure the safety of both mother and infant [6].

The prevalence of PPD in Saudi Arabia has been reported at approximately 14% nationally, with regional studies indicating rates as high as 38.5% in Riyadh [1,7]. These figures are consistent with international reports that show a higher burden of PPD in developing countries, potentially due to cultural, social, and economic stressors [1]. Given the country’s demographic changes, evolving family structures, and the increasing recognition of maternal mental health as a public health priority, it is critical to investigate factors that increase or decrease susceptibility to PPD.

Among the psychosocial factors associated with PPD, social support has consistently been identified as one of the most important protective determinants. Social support refers to the emotional, informational, and practical assistance provided by one’s network, including partners, family, friends, and community members [8]. Adequate support has been shown to buffer the impact of stress, enhance coping capacity, and promote better psychological outcomes for mothers [8,9]. Conversely, low social support is associated with a higher risk of depressive symptoms, poorer maternal functioning, and adverse pregnancy outcomes such as preterm birth and low birth weight [8,10]. Research has also highlighted that unhelpful paternal behaviors or a lack of partner involvement can negatively influence both maternal mental health and infant development [8,11].

Self-efficacy, defined as an individual’s belief in their ability to execute behaviors required to achieve desired outcomes, is another key determinant of maternal mental health [12]. Bandura identified three primary sources of self-efficacy: enactive mastery experiences, vicarious learning (role modeling), and verbal persuasion [13]. In the postpartum context, maternal self-efficacy influences confidence in caregiving tasks, problem-solving, and emotional regulation [10]. Low self-efficacy has been associated with increased vulnerability to depressive symptoms, while higher levels of maternal self-efficacy are linked to more positive parenting practices, improved maternal well-being, and reduced PPD incidence [10,14].

Importantly, social support and self-efficacy appear to be interrelated, with studies suggesting that social support may enhance maternal self-efficacy by providing informational guidance, encouragement, and role models [12]. Similarly, mothers with higher self-efficacy are more likely to seek and utilize available social support, creating a positive cycle that protects against psychological distress [15]. Recent studies have even suggested that self-efficacy may partially mediate the relationship between social support and PPD, highlighting the importance of considering both constructs together when designing interventions [12,15].

Despite growing evidence on postpartum depression (PPD), important gaps remain in understanding how PPD knowledge, perceived social support, and self-efficacy are related to maternal mental health. Recent evidence has emphasized the roles of psychosocial support, maternal confidence, and family and partner involvement in postpartum mental health; however, these factors are often examined separately or within specific intervention settings. In addition, evidence from Saudi Arabia remains limited regarding the combined relationship between PPD knowledge, perceived social support, self-efficacy, and depressive symptoms. This gap is particularly relevant in the Saudi context, where family structures, cultural norms, and gender roles may influence access to information, social support, and maternal coping [16,17]. Adequate knowledge of PPD among mothers, families, and communities may facilitate recognition of symptoms and encourage timely help-seeking [18]. However, knowledge alone may not be sufficient to protect maternal mental health, highlighting the importance of examining psychosocial factors alongside PPD awareness.

**Figure 1:**
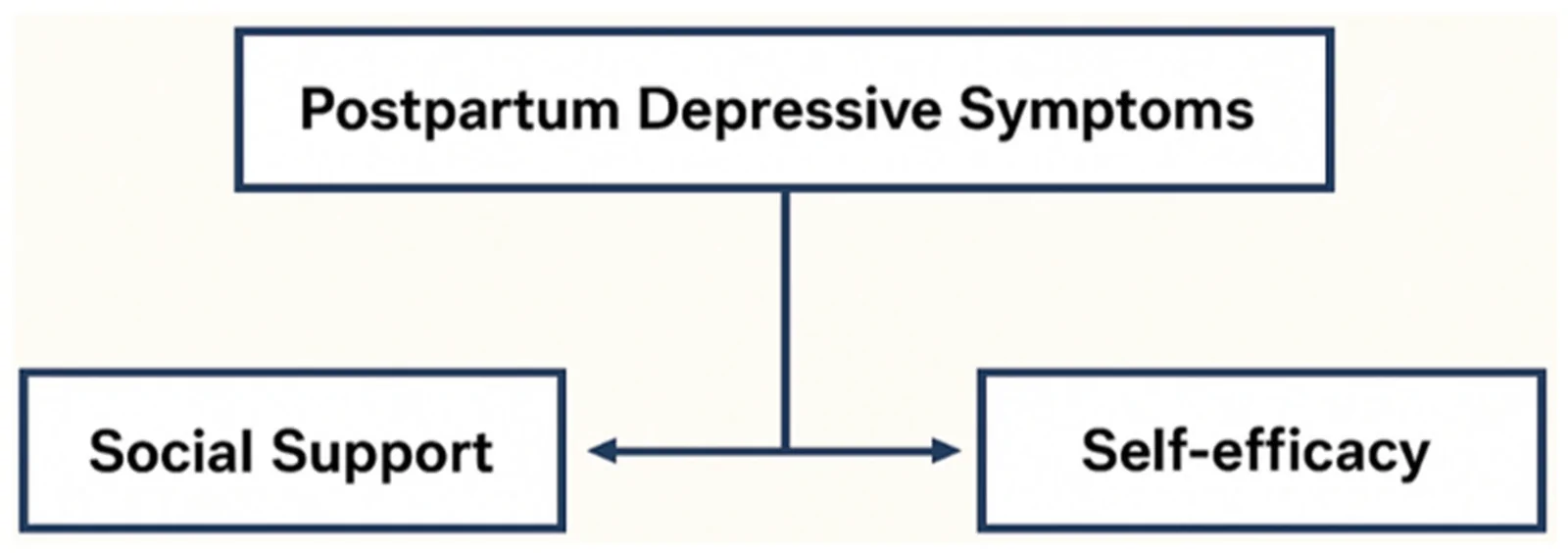
Conceptual associations of self-efficacy and perceived social support with depressive symptoms in the maternal group.

Therefore, this study aimed to address this evidence gap by (1) assessing knowledge of PPD among mothers and participants from the general population, (2) examining the associations of self-efficacy and perceived social support with PPD symptoms among Saudi mothers (Figure 1), and (3) exploring the relationships between selected sociodemographic and psychosocial factors and PPD symptoms. By examining these factors within the same study, the study provides a more comprehensive assessment of PPD-related knowledge and psychosocial factors in the Saudi context and contributes to the limited evidence available from this population.

## 2. Materials and Methods

### 2.1. Study Design

A cross-sectional online survey was conducted to assess PPD knowledge and factors associated with depressive-symptom scores. The study included two groups with different analytical purposes. The maternal group comprised pregnant women and mothers aged ≥18 years and completed measures of PPD knowledge, depressive symptoms, self-efficacy, and perceived social support. The broader-population group completed the PPD knowledge measure and was included to provide an indication of awareness outside the maternal group; it was not a matched comparison group and was not assumed to comprise the actual husbands or relatives of maternal-group participants.

### 2.2. Place and Duration of the Study

The study was conducted online in Saudi Arabia. The duration of the study was three months, starting in January until the end of March 2021.

### 2.3. Study Population and Eligibility Criteria

The study included two participant groups. The first comprised 432 pregnant women and mothers aged ≥18 years. Available reproductive information included number of pregnancies and previous history of PPD. Participants were not excluded on the basis of psychiatric history, medication use, severe medical illness, or obstetric characteristics because these variables were not comprehensively collected. The number of participants who were pregnant versus postpartum and the postpartum interval were not available in the analytical dataset used for this revision; consequently, findings are described as depressive symptoms in a combined maternal group rather than PPD diagnoses. Participants with insufficient questionnaire data were excluded from the relevant analyses, and denominators are reported for each variable.

The second group comprised 275 broader-population respondents recruited to assess PPD knowledge outside the maternal group. It was not designed as a matched comparison group and should not be interpreted as representative of the Saudi general population.

### 2.4. Sampling and Recruitment

A non-probability convenience sampling approach was used for participant recruitment. The required sample size was calculated using the Open EPI website [19], applying the standard formula for a population >10,000 *, which yielded a minimum sample size of 385 participants.

* For population >10,000
n = Z2pq/d2

The study questionnaire was distributed electronically through Google Forms using WhatsApp. The researchers initially disseminated the study invitation through their personal and professional networks and asked recipients to forward the invitation to other potentially eligible individuals. This chain-referral approach enabled recruitment to extend beyond the researchers’ initial networks.

Because the invitation and survey link were redistributed by participants through their own networks, it was not possible to determine the total number of unique individuals who received, viewed, or were eligible to respond to the invitation. Therefore, a conventional response rate could not be calculated. The Google Forms survey was configured to allow only one submission per IP address to reduce duplicate responses.

The use of convenience and chain-referral sampling may have introduced selection bias, as individuals with greater interest in maternal mental health or stronger engagement with the researchers’ networks may have been more likely to participate. Consequently, the sample may not be representative of the wider Saudi population, and the findings should be interpreted within the context of the study population.

### 2.5. Data Collection Tools

Data were collected using structured Arabic-language questionnaires assessing sociodemographic characteristics, knowledge of postpartum depression (PPD), depressive symptoms, self-efficacy, and perceived social support. The questions were reviewed and adapted to ensure that the wording and content were appropriate for the Saudi cultural and social context while maintaining the intended meaning of the original items. The questionnaires were reviewed for clarity, relevance, and comprehensibility before administration.

The Edinburgh Postnatal Depression Scale (EPDS) was used to assess depressive symptoms. Knowledge of PPD was assessed using questions addressing participants’ understanding of PPD, including its symptoms, risk factors, and related characteristics. Self-efficacy and perceived social support were assessed using their respective scales. The Arabic versions of the questionnaires were administered to the participants.

The internal consistency of the study questions was assessed using Cronbach’s alpha. The reliability coefficients obtained in the present study are reported in Section 2.11 (Reliability).

### 2.6. Sociodemographic Characteristics

First section in the mother-group survey included 7 questions, which asked for information about participants’ age, level of education, marital status, family income, number of pregnancies, parental training and previous history of postpartum depression, while the questionnaire that was directed to the general population contained 3 questions, about age, family income and level of education.

### 2.7. Level of Knowledge

PPD knowledge was assessed using eight closed-ended items derived from Afolayan et al. [20]. The questionnaire included multiple choice questions (1 = correct answer, and 0 = wrong answer, I don’t know). Total scores ranged from 0 to 8. Scores of 0–4 were classified as poor knowledge and scores of 5–8 as good knowledge. Because the selected cutoff was study-defined, the resulting categories should be interpreted as descriptive rather than diagnostic.

### 2.8. Edinburgh Postnatal Depression Scale1 (EPDS)

The 10-item Edinburgh Postnatal Depression Scale (EPDS) is an efficient tool to identify risks for PPD [21]. Each item has four response options scored from 0 to 3, resulting in a total possible score ranging from 0 to 30. Items 1, 2, and 4 are scored in the 0-to-3 direction shown, whereas items 3 and 5–10 are reverse scored. The scores for all 10 items are summed to obtain the participant’s total EPDS score, with higher scores indicating greater severity of depressive symptoms. An EPDS score of ≥10 was used to indicate possible depression; the EPDS is a screening instrument and does not establish a clinical diagnosis.

### 2.9. Self-Efficacy Scale

Self-efficacy was assessed using five items selected and adapted from the 10-item General Self-Efficacy Scale developed by Schwarzer and Jerusalem [22]. It utilized a 5-point Likert (1 = strongly disagree, and 5 = strongly agree). Total self-efficacy for each participant was calculated by summing the five question scores. The maximum score was 25 and the minimum score was 5. However, it was divided into three categories. Any participant who scored an average of 5 to 11.6 had low self-efficacy and any participant who scored an average of 11.7 to 18.2 had moderate self-efficacy, while any score of 18.3 or above indicated high self-efficacy.

### 2.10. Social Support Scale

A social support survey was developed by Gregory Zimet [23]. It utilized a 5-point Likert scale (1 = strongly disagree, and 5 = strongly agree). Total social support for each participating was calculated by summing the five question scores. The maximum score was 25 and the minimum score was 5. However, it was divided into three categories; any participant who scored an average of 5 to 11.6 had low social support and any participant scoring an average of 11.7 to 18.2 had moderate social support, while any score of 18.3 or above indicated high social support.

### 2.11. Reliability

Two pilot tests were conducted before distributing the questionnaires to the main samples by recruiting 17 participants for the mothers and 31 participants for the general population. The questionnaire’s reliability was tested by Cronbach’s α, which is used to test the internal consistency of the questionnaire. Cronbach’s α of the Edinburgh Postnatal Depression Scale (EPDS) was (α = 0.8134), for the Self-Efficacy Scale it was (α = 0.8081), for the Social Support Scale it was (α = 0.7716), also for the Knowledge Questions it was (α = 0.7080).

### 2.12. Statistical Analysis

The data was collected, coded and tabulated using John’s Macintosh Project (JMP) version 16 [24], and SPSS version 27 (IBM Corp., Armonk, NY, USA); the alpha error was set to 0.05. Statistical significance was set at two-sided *p* < 0.05. Frequencies and percentages summarize sociodemographic characteristics and knowledge categories. Pearson correlation coefficients quantified bivariate associations of self-efficacy and perceived social support with EPDS scores. A linear regression model examined their simultaneous associations with EPDS scores; regression coefficients are interpreted as associations, not causal effects. One-way ANOVA examined unadjusted differences in mean EPDS scores across sociodemographic categories. Normality and linearity were assessed before regression. Because no multivariable adjustment for clinical and sociodemographic confounders was performed, all inferential findings should be interpreted as exploratory.

## 3. Results

Table 1 represents the sociodemographic characteristics of the sample. Slightly more than one third of the participants were aged 34–41 (34.6%). More than half of them had a bachelor’s degree (59.4%). Most participants were married (94.2%). When it came to number of pregnancies, fewer than half of them had 4–6 children (46.2%). Additionally, 52.5% of the participants had an income of more than 10,000 SR.

The sociodemographic characteristics of the general population (Table 2). The majority of the participants were aged 18–25. Regarding participants’ educational level, 58.1% of them had a bachelor’s degree. Additionally, 61.09% of the participants’ level of income was higher than 10,000 SR.

In total, 73.8% of the mothers had good knowledge of about postpartum depression. On the other hand, 26.2% of them had poor knowledge about postpartum depression. Meanwhile, results on the general population’s level of knowledge about postpartum depression show that 77.5% of them had good knowledge and 22.5% of them had poor knowledge.

Table 3 illustrates the linear regression and correlation between self-efficacy, social support and PPD among Saudi mothers. Self-efficacy (r = −0.246, *p* < 0.001) and perceived social support (r = −0.197, *p* < 0.001) were negatively correlated with EPDS scores. Lower social support and lower self-efficacy will lead to more cases of postpartum depression. Additionally, linear regression analysis was implemented using social support and self-efficacy to predict PPD among Saudi mothers. PPD was entered as dependent variable and variables that had significant correlation with PPD (self-efficacy and social support) were entered as independent variables. Both social support and self-efficacy significantly predicted PPD. Social support had the strongest regression weight on PPD (β = −0.197, *p* = 0.000 **), followed by self-efficacy (β = −0.246, *p* = 0.000 **).

Table 4 shows a comparison of PPD among different sociodemographic variables. It was found that the highest mean PPD was among those who were aged between 18 and 25 (19.5), and the lowest mean was found among those who were aged 58 and above (8.3). This difference is statistically significant (F (5, 421) = 8.1254, *p* = 0.0001 **). It was found that the highest mean PPD was among those with a family income less than 3000 SR (14.8), and the lowest mean was found among those with a family income of more than 10,000 SR (12.2). This difference is statistically significant (F (4, 427) = 3.4265, *p* = 0.009 **).

Mean EPDS scores also differed across educational categories, F (7, 424) = 2.07, *p* = 0.046, and by previous PPD history, F (1, 430) = 73.43, *p* < 0.001. Participants reporting a previous history of PPD had higher mean EPDS scores than those without such a history. These results describe unadjusted group differences and should not be interpreted as independent or causal effects.

## 4. Discussion

### 4.1. Knowledge About Postpartum Depression

The current study found that approximately three-quarters of the participants demonstrated good knowledge of postpartum depression (PPD). This finding suggests a relatively favorable level of awareness among the surveyed participants. However, the proportion identified as having good knowledge should be interpreted in relation to the specific instrument and cutoff used in the present study. A recent study among Saudi women reported that 53.5% had good knowledge of PPD, with knowledge varying according to educational level, occupation, and pregnancy status [25]. Similarly, a study conducted among women in suburban areas reported that PPD is a common condition among postpartum women [26]. The higher proportion observed in the present study may reflect differences in the characteristics of the participants, questionnaire content, scoring criteria, or recruitment settings. Similarly, an Indian study reported adequate PPD knowledge in approximately half of postpartum mothers, demonstrating that substantial variation in PPD literacy exists across populations [27]. The present findings are also consistent with evidence from Malaysia, where most members of postpartum women’s social support networks demonstrated good knowledge of PPD. In that study, parity and other sociodemographic characteristics were associated with awareness, emphasizing that previous reproductive experience may influence familiarity with PPD [28].

In the present study, a relatively large proportion of participants reported multiple pregnancies. Although this may contribute to greater exposure to pregnancy and postpartum-related information, the cross-sectional design does not allow us to determine whether reproductive experience directly improves PPD knowledge. The relatively good knowledge observed in our participants is encouraging because recognition of PPD symptoms and risk factors may facilitate earlier identification and help-seeking. Nevertheless, knowledge alone may not translate into appropriate attitudes or healthcare-seeking behavior. The recent Saudi study by Alsulami et al. demonstrated that although knowledge about PPD was generally good, negative attitudes remained common among Saudi women [25]. A study conducted in Portugal similarly reported an association between the number of children and the level of knowledge about PPD [29]. This distinction is important because improving PPD outcomes requires not only increasing knowledge but also reducing stigma and improving access to appropriate mental health support. However, the present study was cross-sectional, and therefore a causal relationship between reproductive history and PPD knowledge cannot be established. Nevertheless, these findings should be interpreted within the context of the study population and should not be generalized to the wider Saudi population because of the study’s sampling approach and limited sample size.

### 4.2. Postpartum Depression and Social Support

Perceived social support was negatively associated with EPDS scores. Social support may provide emotional validation, practical assistance, shared caregiving, and pathways to professional help, thereby reducing perceived burden. A recent systematic review found consistent associations between greater support and more favorable postpartum psychosocial outcomes [30].

Evidence from Saudi Arabia further supports the relevance of family and social support. A study of women who delivered at King Abdulaziz University Hospital in Jeddah found that family support was significantly associated with postpartum depressive symptoms [31]. More recently, a systematic review and meta-analysis of PPD among Saudi women identified limited family support and poor spouse support among the most consistently reported risk factors, with both demonstrating significant associations with PPD [32]. These findings are particularly relevant to the Saudi context, where family relationships may represent an important source of practical and emotional support during pregnancy and the postpartum period.

Findings from other cultural settings also support the importance of social support in postpartum mental health. A systematic review of studies from Asia found that greater emotional support was significantly associated with a lower risk of PPD, while cultural factors influenced women’s willingness to seek emotional assistance [33]. Similarly, research from Switzerland demonstrated that social and psychosocial circumstances were associated with maternal self-efficacy and postpartum mental health, although these relationships varied across migrant and native-born mothers [34]. Evidence from Canada has also examined social support and maternal self-efficacy alongside postpartum depression, highlighting the importance of psychosocial factors during the transition to motherhood [35]. This is in line with a study conducted in Japan which found that lack of social support from a partner or other relatives showed a higher level of PDD in mothers [9]. Together, these findings suggest that although social support is relevant across diverse settings, its availability, sources, and perceived value may be shaped by cultural and social circumstances.

The present findings should nevertheless be interpreted cautiously. Social support is a multidimensional construct, and perceived support may differ from the actual availability or quality of assistance. In addition, because the present study was cross-sectional, it cannot determine whether inadequate support contributes to depressive symptoms or whether depressive symptoms influence how mothers perceive and report their available support. Longitudinal studies are therefore needed to clarify the temporal relationship between social support and PPD symptoms.

### 4.3. Postpartum Depression and Self-Efficacy

Self-efficacy was negatively associated with EPDS scores. Self-efficacy may be relevant to postpartum mental health because confidence in one’s ability to manage maternal responsibilities may influence coping with the physical, emotional, and practical demands associated with childbirth. Conversely, depressive symptoms may reduce confidence and perceived ability to manage breastfeeding and childcare responsibilities. Recent evidence supports an association between self-efficacy and postpartum depression. A 2023 study conducted among mothers in Saudi Arabia found that higher breastfeeding self-efficacy was inversely associated with PPD, providing context-specific evidence for a relationship between maternal confidence and depressive symptoms [36]. Similarly, a 2024 systematic review and meta-analysis found that postpartum depression was associated with lower breastfeeding self-efficacy, supporting the possibility of a reciprocal relationship between depressive symptoms and maternal self-efficacy [37]. Likewise, a study conducted in a suburb stated that there is a significant association between self-efficacy and PPD [26]. This means that an increase in self-efficacy results in decreasing postpartum depression.

Intervention evidence also suggests that self-efficacy and social support may be modifiable. A 2024 systematic review and meta-analysis of digital parenting interventions found improvements in parenting self-efficacy and social support, together with reductions in depressive symptoms during the transition to parenthood [38]. These findings suggest that interventions aimed at strengthening maternal skills, confidence, and social connectedness may have potential mental health benefits.

The relationship between self-efficacy and postpartum depression has also been observed across different cultural settings. A recent study from Türkiye reported significant relationships between breastfeeding self-efficacy, perceived social support, and EPDS scores, with higher self-efficacy and social support associated with fewer depressive symptoms [39]. In addition, a systematic review and meta-analysis found a significant association between breastfeeding self-efficacy and postpartum depression [37]. Intervention evidence from China further suggests that approaches combining social support with strategies to strengthen maternal self-efficacy may improve psychosocial outcomes [40]. These findings are broadly consistent with the present results and suggest that strengthening mothers’ confidence and support networks may represent relevant components of postpartum mental health care across different cultural settings.

However, the cross-sectional nature of the present study prevents determination of directionality. Higher self-efficacy may be associated with fewer depressive symptoms, but depressive symptoms may also reduce self-efficacy. Therefore, the observed relationship should be considered an association rather than evidence of a causal effect. Longitudinal and intervention-based studies are warranted to determine whether improving maternal self-efficacy can reduce the risk or severity of PPD.

### 4.4. Depressive Symptoms and Sociodemographic Characteristics

Mean EPDS scores differed across age categories in the unadjusted analysis. Younger participants had higher mean scores, but the cross-sectional data do not identify why this difference occurred. Possible explanations include differences in caregiving experience, material resources, social support, or exposure to stress; these mechanisms were not measured and should not be inferred from age alone [41].

Mean EPDS scores also differed across the category of family income. Financial strain may increase stress and restrict access to practical resources, but the present analysis did not measure financial hardship, pregnancy intention, or access to care and did not adjust for confounding. The finding should therefore be interpreted as an unadjusted association rather than evidence that income directly affects depressive symptoms [42].

The findings should also be interpreted within the Saudi cultural context. Extended family structures may provide mothers with practical and emotional support during pregnancy and the postpartum period, potentially strengthening coping and reducing psychological distress. At the same time, family expectations and traditional gender roles may influence how maternal responsibilities are distributed and how women communicate emotional difficulties or seek professional mental health support. Paternal involvement may therefore represent an important but potentially underrecognized component of postpartum support. In addition, religious coping and spiritual practices may serve as important sources of emotional reassurance and resilience for some women. However, these cultural factors were not directly measured in the present study and should therefore be considered possible contextual explanations rather than established mechanisms. Future Saudi studies should specifically examine the roles of family members, fathers, religious coping, and gender-related expectations in relation to PPD.

### 4.5. Education, Reproductive Factors, and Depressive Symptoms

The current finding also revealed that level of education had significant impact on postpartum depression. Previous research suggests that PPD knowledge is influenced by educational and reproductive experiences. In a recent Saudi study, women with a postgraduate education and those working in the health sector demonstrated higher knowledge scores, while non-pregnant women also had greater knowledge than pregnant participants [25]. Another study conducted in Japan found that a lower education level was associated with PPD; the reason behind this could be attributed to the fact that mothers with lower education levels do not often seek professional help [43]. These findings suggest that access to health-related information and educational opportunities may contribute to differences in PPD literacy. It was also found that previous history of depression had a significant effect on PPD, which is similar to the finding of a study conducted in the USA that shows the impact of previous history of PPD [44].

Reproductive experience may also play a role in awareness. A Malaysian study examining PPD awareness among women’s social support networks reported significant associations between awareness and parity [28]. Similarly, studies among pregnant and postpartum women have demonstrated differences in PPD knowledge according to age, education, and occupational status [45]. These findings provide a possible explanation for the association between reproductive characteristics and knowledge observed in the present study. However, such relationships should be interpreted cautiously because knowledge may also be influenced by education, previous exposure to healthcare services, media exposure, and personal experience with mental health conditions.

Importantly, the present findings do not establish that parity or number of children causes better knowledge of PPD. Rather, repeated contact with antenatal, obstetric, and postnatal healthcare services may provide additional opportunities to receive information about maternal mental health. Future studies should therefore examine these relationships using multivariable models that simultaneously account for education, socioeconomic status, healthcare utilization, previous PPD, and exposure to health information.

### 4.6. Limitation

This study has several limitations. First, the use of non-probability convenience and chain-referral sampling may have introduced selection bias and limits the generalizability of the findings to the wider Saudi population. Because the survey invitation was redistributed through participants’ personal networks, the total number of individuals who received or viewed the invitation could not be determined; therefore, a conventional response rate could not be calculated. This recruitment approach may have preferentially reached individuals with greater access to social media or interest in maternal health. Second, the characteristics of the general-population group differed from those of the pregnant women/mothers, and this group was not designed as a matched comparison group; therefore, comparisons between the two groups should be interpreted cautiously.

Third, several potentially important factors associated with PPD knowledge and depressive-symptoms were not comprehensively assessed or included in the analysis, including previous psychiatric history, previous experience of PPD, psychotropic medication use, obstetric complications, access to healthcare, socioeconomic circumstances, marital and family circumstances, and other psychosocial factors. The timing of postpartum assessment may also influence depressive symptom scores and was not fully accounted for in the analysis. Consequently, residual confounding cannot be excluded.

Fourth, the cross-sectional design prevents determination of temporal or causal relationships between the measured variables. Finally, the statistical analyses were primarily descriptive and based on basic tests of association, limiting the ability to simultaneously adjust for multiple potential confounding factors. Further studies using larger, representative samples, standardized assessment of relevant clinical and psychosocial factors, and multivariable statistical approaches are warranted to confirm and extend these findings.

### 4.7. Clinical and Public Health Implications

The findings suggest that PPD literacy, perceived social support, and self-efficacy merit attention in maternal health services. Antenatal and postnatal education could include symptom recognition, stigma reduction, and clear help-seeking pathways. Midwives, family physicians, and primary-care teams may be well positioned to screen with validated instruments and refer women for further assessment. With the mother’s consent, education may also involve husbands and other chosen family supporters to strengthen emotional validation and practical assistance. These are potential applications rather than interventions tested by this cross-sectional study; prospective intervention research is required before effectiveness can be inferred.

## 5. Conclusions and Recommendations

The current study indicated that the majority of the participants had good knowledge regarding postpartum depression. Social support had the strongest regression weight on PPD, followed by self-efficacy. The findings of this study are consistent with the findings of other studies that show that promoting self-efficacy skills is effective in reducing postpartum depression. These findings recommend implementing a program to promote self-efficacy and encourage family members to support mothers during and after pregnancy. Also, further research is encouraged to determine the associated factors that affect PPD in Saudi Arabia.

## Figures and Tables

**Table 1:** Sociodemographic characteristics of the mothers (N = 427).

Characteristics	N (%)	N
Age		427
18–25	17 (3.9)	
26–33	101 (23.6)	
34–41	148 (34.6)	
42–49	98 (22.9)	
50–57	51 (11.9)	
58+	12 (2.8)	
Missing	5	
Level of education		432
Illiterate	1 (0.2)	
Primary school	6 (1.3)	
Secondary	19 (4.3)	
High school	67 (15.5)	
Diploma	53 (12.2)	
Bachelor’s degree	257 (59.4)	
Master’s degree	25 (5.7)	
PHD	4 (0.9)	
Marital status		432
Married	407 (94.2)	
Divorced	20 (4.6)	
Widowed	5 (1.1)	
Number of pregnancies		432
1–3	193 (44.6)	
4–6	200 (46.2)	
7–10	39 (9.0)	
Family income (per month)		432
Less than 3000 SR	13 (3.0)	
3001–6000 SR	45 (10.4)	
6001–8000 SR	58 (13.4)	
8001–10,000 SR	89 (20.6)	
More than 10,000 SR	227 (52.5)	

**Table 2:** Sociodemographic characteristics of general population (N = 275).

Characteristics	N (%)	N
Age		274
10–17	9 (3.2)	
18–25	133 (48.5)	
26–33	40 (14.5)	
34–41	35 (12.7)	
42–49	34 (12,4)	
50–57	17 (6.2)	
58+	6 (2.1)	
Missing	1	
Level of education		275
Illiterate	3 (1.09)	
Primary school	1 (0.36)	
Secondary	11 (4)	
High school	53 (19.2)	
Diploma	18 (6.5)	
Bachelor’s degree	160 (58.1)	
Master’s degree	22 (8)	
PHD	7 (2.54)	
Family income (per month)		275
Less than 3000 SR	12 (4.36)	
3001–6000 SR	30 (10.90)	
6001–8000 SR	24 (8.72)	
8001–10,000 SR	41 (14.90)	
More than 10,000 SR	168 (61.09)	

**Table 3:** Correlations and linear regression coefficients for self-efficacy and perceived social support in relation to EPDS scores.

Variables	r	B	SE B	β	t	*p* Value
Self-efficacy	−0.246	−0.419	0.080	−0.246	−5.262	<0.001
Social support	−0.197	−0.314	0.075	−0.197	−4.171	<0.001

Note: Model R = 0.273; calculated R^2^ = 0.075. B = unstandardized coefficient; SE B = standard error; β = standardized coefficient. *p* values reported as 0.000 in the software output are presented as *p* < 0.001. Statistical significance was defined as *p* < 0.05.

**Table 4:** Unadjusted one-way ANOVA results for sociodemographic variables and EPDS scores.

Variable		Sum of Squares	*df*	Mean Square	*F*	*p*
Age	Between Groups	1128.519	5	225.704	8.1254	<0.001
	Within Groups	11,694.348	421	27.778		
	Total	12,822.867	426			
*Family income (per month)*	Between Groups	405.427	4	101.357	3.427	0.009
	Within Groups	12,630.738	427	29.580		
	Total	13,036.164	431			
*Level of education*	Between Groups	430.544	7	61.506	2.069	0.046
	Within Groups	12,605.620	424	29.730		
	Total	13,036.164	431			
History of PPD	Between Groups	1901.331	1	1901.331	73.425	<0.001
	Within Groups	11,134.834	430	25.895		
	Total	13,036.164	431			

Statistical significance was defined as *p* < 0.05.

## Data Availability

Data available on request due to restrictions, due ethical reasons as the data is own by Princess Nourah bint Abdulrahman University.

## Abbreviations

The following abbreviations are used in this manuscript:

EPDS	Edinburgh Postnatal Depression Scale
PPD	Postpartum depression
NA	Not applicable
